# Comparison of azithromycin and erythromycin in the treatment of mycoplasma pneumonia in children

**DOI:** 10.12669/pjms.36.2.1441

**Published:** 2020

**Authors:** Rui Han, Qianqian Yu, Guohui Zhang, Baoqiang Li, Shuzhen Han, Guiying Li

**Affiliations:** 1 Rui Han, Department of Pediatrics (I), Binzhou People’s Hospital, Shandong, 256610, China; 2 Qianqian Yu, Department of Respiration, Binzhou People’s Hospital, Shandong, 256610, China; 3 Guohui Zhang, Department of Pediatrics (II), Binzhou People’s Hospital, Shandong, 256610, China; 4 Baoqiang Li, Department of Pediatrics (II), Binzhou People’s Hospital, Shandong, 256610, China; 5 Shuzhen Han, Neonatal Intensive Care Unit (NICU), Binzhou People’s Hospital, Shandong, 256610, China; 6 Guiying Li, Department of Pediatrics (I), Binzhou People’s Hospital, Shandong, 256610, China

**Keywords:** Azithromycin, Erythromycin, Children, Clinical efficacy, Mycoplasma pneumonia

## Abstract

**Objective:**

To study and compare the clinical effects of azithromycin and erythromycin on children with mycoplasma pneumonia.

**Methods:**

Total 132 children with mycoplasma pneumonia who were admitted to our hospital between November 2017 and September 2018 were selected as the research subjects. All the children were divided into an observation group and a control group according to random number table, 66 each. The observation group was treated with azithromycin, while the control group was treated with erythromycin. The therapeutic effect, incidence of adverse reactions and disappearance time of clinical symptoms were compared between the two groups.

**Results:**

The total efficacy of the observation group was 98.04%, and that of the control group was 74.51%; there was a significant difference (X^2^=7.184, P=0.007). The incidence of adverse reactions in the observation group was 15.69%, significantly lower than that in the control group (41.18%) (X^2^=6.376, P=0.002). The disappearance of fever, cough, rale and X ray shadow of the observation group was significantly earlier than that of the control group, and the difference was statistically significant (P<0.05).

**Conclusion:**

Compared with erythromycin, azithromycin is more effective in the treatment of mycoplasma pneumonia in children. Azithromycin can further shorten the improvement time of clinical symptoms and signs and has few adverse reactions and high safety. It is worth clinical application.

## INTRODUCTION

Mycoplasma pneumonia is a common respiratory disease caused by mycoplasma pneumoniae.^1^,^2^ It is reported that the main clinical manifestations of mycoplasma pneumonia include fever, expectoration, anorexia, headache, sore throat, etc. However, it has been found that the clinical symptoms of mycoplasma pneumonia in patients are related to the age of patients, and the smaller the age, the more atypical the clinical symptoms of mycoplasma pneumonia.^3^,^4^

At present, the pathogenesis of mycoplasma pneumonia in children has not been clearly defined clinically, but it is considered being related to autoimmunity and humoral immunity of children.^5^,^6^ Therefore, the clinical treatment of mycoplasma pneumonia in children is mainly drug therapy. At present, the most commonly used drugs are macrolide antibiotics. Azithromycin and erythromycin are macrolide antibiotics, which have good inhibitory effect on mycoplasma.^7^,^8^ This study compared the clinical efficacy of azithromycin and erythromycin through treating children with mycoplasma pneumonia, with the intention of providing a reference for clinics.

## METHODS

One hundred and thirty-two children with mycoplasma pneumonia who were admitted to our hospital between November 2017 and September 2018 were selected as the study subjects. The calculation of the sample size followed the following formula:


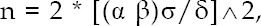


where δ represent the required distinction degree, represents the overall standard deviation or its estimated value s, α and β and can be checked out from the row of degree of freedom v ═ ∞ - in the table of t critical value.

The diagnostic criteria followed was of mycoplasma pneumonia in children in Practical Paidonosology. The inclusive criteria were conforming to the diagnostic criteria of mycoplasma pneumonia in children, receiving no treatment of hormone and antibiotics, and having no other extrapulmonary complication. They were divided into a control group and an observation group according to random number table method, 66 each. The control group consisted of 21 males and 45 females, aged from one to 15 years, with an average age of (7.9±2.4) years; the duration of mycoplasma pneumonia was 2 to 11 days (average (7.16±1.14) days. There were 31 males and 35 females in the observation group, aged from two to 14 years, with an average age of (8.3±2.6) years; the duration of mycoplasma pneumonia was 3 to 12 days (average (7.85±1.94) days). There was no significant difference in the general data such as sex and age between the two groups (P>0.05); therefore, the results of the two groups could be compared. This study was approved by the ethics committee (Ref. No. 134, dated May 8, 2019) of our hospital. All the family members of the selected children signed informed consent Form.

### Therapeutic methods

All the children were given the same conventional treatment, including cough relief, phlegm resolving, heat clearing, etc. The control group was treated with erythromycin (Beijing Shuanghe Pharmaceutical Co., Ltd., China; State Food and Drug Administration approval number: H11021588) on the basis of the conventional treatment. Firstly, the patients were treated by intravenous injection of 20 mg/kg erythromycin and 200 mL of glucose solution, once a day. After the disease condition became stable, the patients were given oral administration of 100 mg/kg erythromycin for suspension (State Food and Drug Administration approval number: H10970222), twice a day, for two weeks. The observation group was treated with azithromycin in addition to the conventional treatment. Firstly, they were treated by intravenous injection of 100 mg/kg azithromycin (State Food and Drug Administration approval number: H20064098) and 200 mL of glucose solution, once each day. After the disease condition became stable, the patients were given oral administration of 5 mg/kg azithromycin granules, twice a day, for two weeks.

### Observation indicators

1. The clinical efficacy of the two groups was observed. The evaluation criteria of curative effect were as follows.^9^ Efficacy was considered as significant if all the clinical symptoms completely disappeared, the vital signs completely disappeared, and the X-ray chest film examination indicated no symptoms of pneumonia. Treatment was considered as effective if the clinical symptoms relieved significantly, the vital signs significantly relieved, and the X-ray chest film examination indicated mild symptoms of pneumonia. Treatment was evaluated as ineffective if the clinical symptoms remained unchanged or even aggravated. Total effective rate = (number of significantly effective cases + number of effective cases)/total number of cases × 100%.
2. The disappearance time of clinical symptoms of the two groups after treatment was observed.
3. The occurrence of adverse reactions in the two groups was observed and recorded.

### Statistical analysis

SPSS20.0 was used to analyze and process the data. Measurement data was expressed as Mean±Standard Deviation and processed by t test. Counting data was expressed by % and processed by X^2^ test. Difference was considered statistically significant if P<0.05.

## RESULTS

The total effective rate of the observation group was 95.5%, significantly higher than that of the control group (81.8%) (P<0.05, Table-I).

**Table-I:** Clinical efficacy between the two groups (%).

Group	Observation group	Control group	X^2^	P
Markedly effective	38(57.6)	26(39.4)	/	/
Effective	25(37.9)	28(42.4)		
Ineffective	3(4.5)	12(18.2)		
Total effective rate	63(95.5)	54(81.8)	7.184	0.007

The time of fever abatement, cough disappearance, rale disappearance and X-ray shadow disappearance in the observation group was significantly shorter than that in the control group, and the difference was statistically significant (P<0.05, Table-II).

**Table-II:** Disappearance time of symptoms and signs between the two groups [(Mean ±SD), d].

Group	Observation group	Control group	t	P
Antipyretic time	2.88±1.25	5.72±1.54	9.218	0.001
Cough disappearance time	9.02±1.97	12.33±3.72	5.634	0.014
Disappearance time of rale	7.25±2.46	11.13±3.48	6.517	0.005
Disappearance time of X-ray shadow	3.22±1.01	6.98±1.56	13.372	0.000

The incidence of adverse reactions in the observation group was 10.6%, significantly lower than that in the control group (25.0%) (P<0.05, Table-III).

**Table-III:** Occurrence of adverse reactions between the two groups during treatment (%).

Group	Observation group	Control group	X^2^	P
Local pain	2(3)	7(10.6)	/	/
Rash	1(1.5)	4(6.1)		
Gastrointestinal reaction	4(6.1)	5(7.6)		
Liver function damage	0(0)	1(1.5)		
Incidence of adverse reactions	7(10.6)	17(25.8)	6.376	0.002

## DISCUSSION

Mycoplasma pneumoniae infection is the main pathogenic factor of mycoplasma pneumonia in children. A study showed that about 9.6%~66.7% of lung infections were caused by mycoplasma pneumoniae.^10^ Mycoplasma pneumonia in children is a common respiratory tract disease, and its incidence shows an increasing tendency in recent years.^11^,^12^

Considering that children with mycoplasma pneumonia are in the stage of physical development, appropriate antibiotics should be selected in the course of treatment. Macrolide antibiotics are the primary drugs for the treatment of mycoplasma pneumoniae infection. Erythromycin is a common drug for the treatment of mycoplasma pneumonia and is also the most commonly used drug in grass-roots hospitals.^13^ Erythromycin can effectively alleviate the clinical symptoms of children, reduce lung shadow and shorten the course of disease. However, a study points out that erythromycin is unstable in acidic environment and easy to have problems such as decomposition, which will lead to gastrointestinal adverse reactions.^14^ Moreover, erythromycin generally has large dose, slow infusion and long course. The long-term intravenous injection can easily cause pain on the puncture site and phlebitis, and many children cannot adhere to the treatment, resulting in poor compliance.

Azithromycin is a new macrolide antibiotic, which can block the synthesis of proteins by fully combining the ribosome subunits of pathogens to achieve the purpose of antimicrobial activity. In the treatment of mycoplasma pneumonia in children, azithromycin has the following advantages:^15^-^17^ 1) it has a broad spectrum of antimicrobial agents, i.e., it can inhibit and kill most Gram-negative and positive bacteria; 2) it has good tissue permeability, i.e., it can quickly reach the infected site and enhance the drug concentration of the infected site; 3) it has a long half-life of plasma drug, i.e., more than 40 days; the stability of azithromycin is 300 times higher than that of erythromycin, which can reduce the number of medications and improve the compliance of children.

Clinical effects of azithromycin and erythromycin on children with mycoplasma pneumonia were compared and analyzed in this study. The results showed that the total effective rate of the observation group was 95.5%, which was significantly higher than that of the control group (81.8%), The result was similar to the results of Yang.^18^ In addition, in the study of Tang,^19^ 90 children with mycoplasma pneumonia were selected and randomly divided into a control group and a research group. They were treated with intravenous infusion of erythromycin and sequential therapy of azithromycin respectively. The results showed that the disappearance time of cough and rale in the research group was significantly shorter than that in the control group, which was also confirmed by the results of this study. In this study, the results showed that the disappearance time of fever, cough, rale and X-ray shadow of the observation group was significantly shorter than that of the control group, suggesting that the clinical effect of azithromycin was more significant. Moreover, the incidence of adverse reactions in the observation group was significantly lower than that in the control group, which indicated that azithromycin was safe, similar to the previous research results.^20^,^21^ The reason was that the chemical structure of azithromycin was more stable and its half-life was long, which was helpful to reduce the symptoms appeared in the treatment process.

## CONCLUSION

In conclusion, azithromycin is more effective than erythromycin in the treatment of mycoplasma pneumonia in children. It can significantly shorten the disappearance time of clinical symptoms and has less adverse reactions and high safety. It is worth further clinical application.

### Authors’ Contribution:

**RH & QQY:** Study design, data collection and analysis.

**RH, QQY, GHZ, BQL & SZH:** Manuscript preparation, drafting and revising.

**RH & GYL:** Review and final approval of manuscript, are responsible for integrity of research.
